# The growing caseload of chronic life-long conditions calls for a move towards full self-management in low-income countries

**DOI:** 10.1186/1744-8603-7-38

**Published:** 2011-10-10

**Authors:** Josefien van Olmen, Grace Marie Ku, Raoul Bermejo, Guy Kegels, Katharina Hermann, Wim Van Damme

**Affiliations:** 1 Department of Public Health, Institute of Tropical Medicine, Nationalestraat 155, Antwerp, 2000, Belgium; 2 Veterans Memorial Medical Center, North Avenue, Diliman, 1100, Quezon City, Philippines; Department of Public Health, Institute of Tropical Medicine, Nationalestraat 155, Antwerp, 2000, Belgium; 3 Provincial Health Office, Capiz Provincial Government, Philippines; Department of Public Health, Institute of Tropical Medicine, Nationalestraat 155, Antwerp, 2000, Belgium; 4 Médecins sans Frontières, Thyolo, Malawi

## Abstract

**Background:**

The growing caseload caused by patients with chronic life-long conditions leads to increased needs for health care providers and rising costs of health services, resulting in a heavy burden on health systems, populations and individuals. The professionalised health care for chronic patients common in high income countries is very labour-intensive and expensive. Moreover, the outcomes are often poor. In low-income countries, the scarce resources and the lack of quality and continuity of health care result in high health care expenditure and very poor health outcomes. The current proposals to improve care for chronic patients in low-income countries are still very much provider-centred.

The aim of this paper is to show that present provider-centred models of chronic care are not adequate and to propose 'full self-management' as an alternative for low-income countries, facilitated by expert patient networks and smart phone technology.

**Discussion:**

People with chronic life-long conditions need to 'rebalance' their life in order to combine the needs related to their chronic condition with other elements of their life. They have a crucial role in the management of their condition and the opportunity to gain knowledge and expertise in their condition and its management. Therefore, people with chronic life-long conditions should be empowered so that they become the centre of management of their condition. In full self-management, patients become the hub of management of their own care and take full responsibility for their condition, supported by peers, professionals and information and communication tools.

We will elaborate on two current trends that can enhance the capacity for self-management and coping: the emergence of peer support and expert-patient networks and the development and distribution of smart phone technology both drastically expand the possibilities for full self-management.

**Conclusion:**

Present provider-centred models of care for people with chronic life-long conditions are not adequate and we propose 'full self-management' as an alternative for low-income countries, supported by expert networks and smart phone technology.

## Background

The problem of chronic diseases has risen up the agenda of global health policy makers in recent years [1-6]. The growing numbers of patients with Chronic Life-Long Conditions (CLLC), such as diabetes and hypertension, puts an immense burden on health systems and populations, because of increased needs for health care providers and steadily rising costs of health care services.

The present response of health systems, both in high and in low income countries, is highly inadequate. The professionalised models of chronic care that have been developed in high income countries are labour-intensive and very costly and therefore unsustainable [7-9]. In addition, these models fail to show substantial improvements in risk factor control and health outcomes for chronic patients [10-12]. In Low-Income Countries (LIC), the scarce resources and the lack of quality and continuity of health care result in high expenditure and very poor health outcomes for people living with CLLC.

The failure of health systems to respond adequately to the needs of patients with CLLC, especially in LIC, is generally acknowledged. The proposals to improve this response highlight the need to strengthen primary care as a way to manage the problem and focus on the development of essential packages of care and prevention for specific (groups of) chronic conditions [13-15]. The question is whether these suffice. Are these provider-centred approaches feasible and sustainable in the context of a steadily increasing caseload of patients? Are they even logical from the perspective of people with CLLC?

The aim of this paper is to show that present provider-centred models of care for people with CLLC are not adequate and to propose an alternative for LIC in the form of 'full self-management', supported by external resources in the form of expert patient networks and smart phone technology. This alternative model is inspired by the nature of CLLC and current opportunities presented by the growing experience with expert patients support groups and the wave of innovations in communication and information technology.

## Discussion

### The burden of chronic conditions

There are various understandings of 'chronic diseases' in the literature and plenty of terms are being used. Many people use the word chronic conditions mainly to refer to non-communicable diseases (NCD) [16,17]. Other people use a very broad definition, for instance including long-term but temporary diseases and physical or mental handicaps [15,18,19]. Neither the very broad definitions of chronic conditions nor the focus on NCD highlight the specificities of long-term conditions for the organisation of health care systems. We therefore propose to use the term 'Chronic Life-Long Condition' (CLLC) and define it as 'life-long conditions requiring long-term medical interventions and adherence to medication and adjustments in life'. This includes a range of non-communicable and communicable diseases, but explicitly excludes diseases that can be cured and thus are of a temporary nature, such as tuberculosis.

Many diseases qualify for being labelled CLLC and their importance varies per continent and even between areas in the same country. The particular endemic situation of each context should determine local priorities. At a global level, the burden of chronic diseases is primarily caused by hypertension & cardiovascular disease, diabetes, chronic lung diseases, mental illness and, particularly in sub-Saharan Africa, by HIV/AIDS [15,20]. The major burden is in low and middle income countries, which are estimated to account for 80% of the global mortality related to chronic disease [19]. The prevalence of the important risk factor hypertension is highest in low and middle income countries [21]. Abegunde et al. calculated that chronic diseases were responsible for around 50% of the total disease burden in 23 selected low and middle income countries and showed that the age-standardised death rates for chronic diseases were higher than in high income countries [22]. An additional problem is the co-morbidity of CLLC. Several studies show a 30 to 60% prevalence of hypertension among diabetes type 2 patients and many diabetes patients die of cardiovascular complications [23,24]. The development of diabetes among AIDS patients on Anti-Retroviral Treatment (ART) is also well described [25].

By their nature, CLLC persist over time and this leads to an ever-increasing caseload of patients. Estimates are that at present, 972 million people are living with hypertension and cardiovascular diseases, 285 million with diabetes and 33 million with HIV/AIDS [20,26-28]. More than 50% of all these cases are in LIC. The numbers of people suffering from CLLC will continue to rise, because of increasing incidence, ageing and, partly, because better treatment can keep people alive longer. This will lead to an increasing need for health care. The use and consumption of medication and medical technology will rise, and will thus increase the overall health cost for societies, families, and individuals.

The current reaction of health systems is inadequate. The professionalised models of chronic care that have evolved in western countries rely heavily on professional care, specialized staff, and medical technology. These models are not sustainable, because they will lead to an escalation of cost and because staff to deliver the care will not be available. The direct health care cost for people with chronic conditions in the United States (US) accounts for three quarters of their national health care expenditure and the projected total cost for chronic diseases in 2023 amounts to 4.2 trillion US dollar [7,29-31]. In European countries, the cost of diabetes care is reported to be between 2 and 15% of national health expenditures [10]. Health care providers have great difficulties to deal with the increasing workload and to provide access and continuity of care for chronic patients [8]. In the United Kingdom, people with CLLC account for 80% of all visits to general practitioners [32]. Stress and low job satisfaction are widespread phenomena among health care personnel [8]. The combination of increasing health care demands and decreasing graduates in general practice will result in an estimated shortage of 27% of general practitioners by 2025 in the US [33].

Moreover, the results of health professional-led care for CLLC are not impressive. Nolte et al. observed that "chronic conditions frequently go untreated or are poorly controlled until more serious and acute complications arise. Even when recognised, there is often a large gap between evidence-based treatment guidelines and current practice" [10,12]. In the US, the "majority of patients with hypertension, diabetes, tobacco addiction, hyperlipidaemia, congestive heart failure, chronic atrial fibrillation, asthma, and depression are inadequately treated" [29]. For instance, only one third of patients with hypertension under pharmacological treatment achieve the recommended blood pressure goals, more than half of diabetic patients have hemoglobin A1 c levels above the recommended target of 7.0% [34,35]. A review of quality of clinical care in Australia, New Zealand and the United Kingdom found that even the best-performing practices failed to perform routine examinations and cardiovascular risk control in half of their diabetes patients [12]. The failure in secondary prevention leads to substantial expenses for long-term complications, such as coronary heart disease and end-stage renal disease [34].

Various western countries have made efforts to cope with the specific requirements of chronic conditions and introduced chronic disease management programmes and health care that is not based on an 'acute care model' [10,36,37]. The most widely known model is the Chronic Care Model (CCM), which acknowledges that a big portion of chronic care takes place outside the formal health care system, focusing on linking active people with chronic conditions with pro-active teams of health professionals [38]. Although this is an attractive conceptual model and evidence shows that it can improve quality and outcomes of care, it is still very resource intensive and difficult to implement [39,40]. The focus often tends to remain on the organisation of the professional providers [40]. The adapted model for low resource settings, the 'Innovative Care for Chronic Conditions' framework, has not yet been described in practice [18].

In LIC, health care is still very much episodic, that is, focusing on acute care for health events for a limited period of time [3,15,41-44]. Other barriers are related to the general malaise of health systems such as a maldistribution of adequately trained and well-motivated staff and a lack of affordable and reliable drugs and diagnostics [45]. The poor quality of care stimulates chronic patients to continue to search for care from various providers, also called health care shopping. The cost for this care is mostly paid out of pocket. This often induces catastrophic health care expenditure. The health outcomes for patients with CLLC in LIC are very poor. In 2005, the age-standardised death rates for chronic diseases were estimated to be 54% higher for men and 86% higher for women than those for men and women in high-income countries [22].

The only CLLC for which universal coverage of treatment has been pursued on a large scale in LIC is HIV/AIDS. Although the scaling-up of ART has led to coverage rates of up to 60% in some countries, the experience with cohorts of patients on treatment also shows how difficult it is to retain patients in treatment and to reach good results [46]. Moreover, the sustainability of the delivery models for ART has already been put into question; it seems thus rather unrealistic to expect that there will be massive donor funding available for patients with other CLLC in these countries in the coming years [47].

We thus conclude that the current response of health care systems to the burden of CLLC has failed, especially in LIC. The ongoing discussion on the organisation of care for CLLC highlights the need to strengthen primary care as a way to manage the problem and focuses on the development of essential packages of care and prevention for specific (groups of) chronic conditions [13-15]. We argue that if health systems, especially in LIC, want to respond adequately to the needs of patients with CLLC, they will have to invent delivery models that rely less on qualified personnel and that are much cheaper than the present models.

### Characteristics of Chronic Life-Long Conditions and their Management

The definition of CLLC highlights the fact that people face a condition that will not disappear and have to make adjustments in their life to remain as healthy as possible. We will discuss some important characteristics of CLLC and their implications for the organisation of health care.

First, people with a CLLC are not only patients, but also people with a 'normal' life. They have needs that are related to their CLLC, but they also typically have a family and social life, employment, hobbies, likes and dislikes and ambitions. When they are diagnosed with a CLLC, they have to 'fit' this new and rather unpleasant element into the rest of their lives. Medical sociologists introduced the term 'biographical disruption' to denote the impact that the experience of chronic illness has on persons' everyday life and described the process of incorporating chronic illness into life and identity, both in terms of cognitive processes as in practical responses, for instance by mobilising of social, physical, medical and cultural resources [48,49]. We use the term 'rebalancing', to emphasise the dynamic exercise - or, if one prefers, a 'juggling act' - that people with CLLC face for the rest of their lives. For instance, persons who are diagnosed with diabetes will have to adjust their eating pattern and get used to regular glucose monitoring and medication. Due to these requirements, jobs and behaviour at parties will be affected, to name just a few aspects in life that are bound to change.

Second, the patients themselves play a crucial role in the management of their CLLC. The greater part of living with a chronic condition takes place without external support. Many challenges, unforeseen problems and questions occur at the 'in-between moments' of the contacts with the health care providers. Patients continuously make decisions that influence the course of their disease, on adherence to medication, healthy behaviour and coming for follow-up visits [50].

Third, the long duration of the condition and the relatively big influence patients have on it are an opportunity for patients to gain knowledge and expertise in their condition and its management [37]. In the course of their CLLC, many gradually become 'experts' in how to live with the disease; some gain truly amazing expertise as 'experience experts' [51].

The characteristics of CLLC as described above have implications for the design and organisation of health care. It becomes apparent that what patients need is an extended web of support that will facilitate most of the coping and life adjustment processes that they need to undertake to gain mastery of their chronic condition [52]. Health care, including access to medications, diagnostics and professional advice, remains of course an important strand in this web.

In addition, the crucial role and growing expertise of people to manage their own conditions in combination with the other aspects of life changes the traditional hierarchic 'doctor-patient' relationship into a relationship that is more 'equal'[37,53]. The health care provider has expert knowledge about the disease in general. But patients are experts in living with the disease, in how the disease changes their lives and in gaining some control over the course of their illness. They know how to interpret physical signs and the reaction of their body to changes in behaviour or medication.

Moreover, patients know what happened in their own lives in the periods between contacts with a health care provider and how that could have influenced their current condition. They know whether they took the medication, whether they followed the lifestyle recommendations and whether or not they had had a stressful time. Patients are not likely to share all this relevant information with the health care provider at the moment they see him, for several reasons. There is limited time in a consultation, they might not remember or deem important everything, or, they might decide not to tell the doctor, because they do not want to. For instance, intensive counselling about an adequate lifestyle can induce patients to give socially desirable answers rather than tell the truth, trying to meet expectations and goals set by the provider [54,55]. Studies about people with chronic conditions show that "they attempt to fit in with normative, biomedical expectations of correctness" [56]. In order not to disappoint their provider or to be reprimanded, they might adapt their behaviour in the period just before the appointment with their health provider to make sure their parameters (blood sugar, blood pressure, etc.) are within acceptable limits. In general, health providers will assess the condition of their patients based upon their (incomplete) stories, complement it with their own (implicit) assumptions and base their treatment decision on this combination [57]. This situation describes a reverse asymmetry of information: the patient has more information relevant to the decision at stake than the health provider. This may partly explain the poor results of treatment of CLLC even in health care organisations adapted to chronic care in high-income countries. The health provider just does not have enough information to make the best treatment decision.

### Towards full self-management

From the above, we can conclude that the present response of health systems to CLLC is failing. Firstly, professionalised chronic care models rely heavily on qualified personnel and are very expensive [30,31]. Exporting these models to LIC where qualified human resources are scarce and health systems are underfunded is not the right way forward. Secondly, provider-centred models of care for patients with CLLC globally deliver poor results, because they ignore the nature of chronicity and do not adequately reconceptualise the role of the patient [29,37,51]. However, we think there are new approaches possible that may be more sustainable.

**Figure 1:**
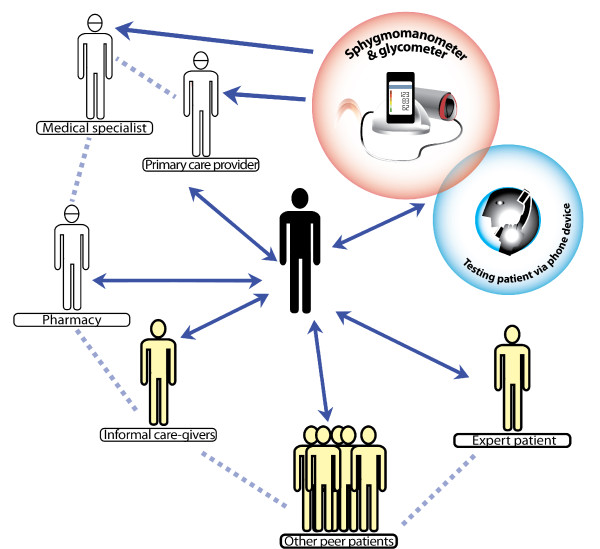
**"The person living with a Chronic Life-Long Condition as the hub of disease management, supported by smart phone technology, peer support, and other resources, including a primary care provider and informal care givers"**.

We propose 'full self-management', the elements of which are shown in Figure 1. People with CLLC should become the management hub of their own care. They should not only be at the centre of receiving care, but take full responsibility for their chronic condition. In this, they need to be supported by peers, immediate care-givers and families, by information and communication tools, by medication and technology and by professionals for specialised advice, information, certain tests and diagnosis. This is intended as the ultimate empowerment of patients: individuals or groups living with a CLLC gain mastery over their own affairs, by an increasing capacity to make choices and to transform those choices into desired actions and outcomes [58].

The literature about patient empowerment and self-management already brought about a major paradigm shift, from a so called 'traditional medical' to a collaborative patient-doctor model [50]. Our full self-management acknowledges the value of this model, but adds other resources than the professional to it. The patient does not only have contact with a limited number of professionals but is the centre of a web (the hub) in which he has the disposal of many resources, from family, peer patients, to technological devices that help him to master his own daily disease management. The extending range of tools and information available makes the realization of this concept more possible. Table 1 shows the differences between three models of care - 'traditional medical care', patient-professional partnership and full self-management.

**Table 1:** Differences between different models of care (adapted from [29])

Issue	Traditional medical care	Patient-Professional Partnership	Full self management
**Relationship patient - health professional**	Professionals as experts - patients as passive	Shared expertise and two way relationship: professionals as disease experts - patients as 'life' experts	Patient as centre of a web linked with many other resources of which the professional is one

**Locus of Control**	Professional	Patient and professional, shared responsibility	Patient, supported by professionals and other resources

**Problem identification**	Professional: medical perspective	Patient: illness perspective	Patient: rebalancing perspective

**Problem solving**	By professional	By patient, helped by professionals' **teaching**	By patient, helped by professionals, peers, technology (**mastering**)

**Technical resources**	Focused on professional	Focused on professional	Focused on patient

**Behaviour influence through**	External motivation	Internal Motivation	Motivation via different channels, internal and external

How can full self-management for people living with CLLC be realised? A change in the traditional provider-patient relationship is needed. The empowerment of the patients should be the ultimate aim of 'care'. Frequently mentioned obstacles to this goal are socio-economic barriers, patient comorbidities and complex treatments and the organization of practice of the health care provider. On both sides, the "pervasive socialization of both patients and practitioners to the traditional medical model of care" is probably an even greater impediment [52,59]. In an earlier paper, we recognised the challenges and gave suggestions of how primary care providers can contribute to the empowerment of patients [60]. Doctors need to have confidence in the competence of patients and should change their way of thinking in terms of responsibility, from being 'responsible for' towards 'responsible to' their patients. Many patients, on the other hand, need to learn to adjust their expectations of the role of the doctor in their life, to gain confidence and take responsibility for their own health. The design of health systems should be adjusted to support and motivate both patients and care providers in these processes. This means the redirection of financial resources to primary care providers and to people with CLLC, to develop and maintain coordination, communication, technical tools and other resources to stimulate and reinforce self-management. For instance, insurance systems with personal budgets for persons with CLLC could enable them to assemble a personal self-management support package existing of communication tools, practical support and information materials. The 'consumer directed services' approach applied in a number of western countries is an example of how it can work. This approach is "designed to maximise the autonomy and independence of persons with physical dependencies by giving them greater choice and control over personal care and other in-home services and providers" and empowers persons with CLLC "to assume responsibility for key decisions, including assessment of their own needs, determination of how and by whom needed services should be delivered, and monitoring the quality of services received" [61]. Other suggestions to adapt systems to a more empowering approach are the involvement of people with CLLC and their carers in the design of the systems, for instance by patient advisory bodies, changes in staff training, recruitment and valorisation, to encourage an empowering attitude towards patients and the development of tools assisting people with CLLC to articulate their goals, preferences and expectations from their own perspectives [61,62].

The attitude towards illness and (chronic) disease management is also determined by wider socio-economic determinants that influence access to care, self-perceived effectiveness and the capacity to mobilise resources. This is an imperative for health system to guarantee optimal access to care and to self-management support and to address wider social determinants of health.

There are important opportunities that can accelerate the shift towards full self-management. The increased availability of information and communication tools creates endless possibilities for networks and targeted contacts. Due to the development of technology, new tools for diagnostics and management become available. Over time, they will also get cheaper. We will discuss two recent developments in more detail, because they are real 'game changers' in the relationship between health care providers and people with CLLC. The first one is the large number of people who have become experts in living with a CLLC. The second one is smart phone technology.

### Patients supporting patients

In the last decade, patients themselves have become a resource for care and support to other patients. There has been a growth of peer support networks, programmes and similar initiatives that aim to capitalise on the expertise of patients [63]. They are so-called 'expert patients', 'peer educators' and likewise. Peer support is the provision of support from an individual with experiential knowledge based on sharing similar life experiences [64].

The content of these initiatives varies. They are developed within a specific context and in reaction to the presence or absence of health care services. In high-income countries, they are usually part of a wider chronic care programme, focus on education, and are facilitated by professionals [65]. Publicised examples include the diabetes support groups in New Zealand, [66]; and the patient led self-management for people with chronic arthritis and other chronic diseases [67,68]. In areas where professional health care systems cannot or do not deliver appropriate care, patients can also be involved in other tasks, such as follow-up of patients or delivery of treatment [47,69,70]. Most publications from LIC in this field are so far related to HIV/AIDS programmes, many focusing on behavioural peer programmes [71], but recently also about peers involved in the delivery of anti-retroviral treatment [72-74]. Peer networks for other chronic diseases, especially diabetes, get increasing attention [75-80]. We have found publications about diabetes peer projects in Jamaica [81] and Indonesia [63].

From the perspective of patients with CLLC, the concept of peer support is very powerful. Some people have become experts in rebalancing their life, in combining disease management with all other aspects of life. Health care providers, on the other hand, are mostly only experts in one aspect, like medical treatment or diet. Fellow patients are more likely to influence the perception of self-efficacy and confidence of peer patients helping them to change behaviour and to cope with their disease [62]. Moreover, the empathy that peer educators may possess enables them to better reach out to their fellow patients. However, empathy and experience do not automatically result in adequate support and advice to others. They need adequate direction.

The results of peer-support programmes are mixed. The most obvious effect is that of social support and improved well-being among patients. Several studies show a positive impact on behaviour change. There is no strong evidence for improved health outcomes for patients [68,82]. Like many other lay-worker and voluntary programmes, peer support programmes suffer from problems with quality, motivation and sustainability. The participation of people in such programmes depends on their focus and design [67,83]. Expert patients, in order to become valuable peer educators, need to be adequately trained and supported. This will entail considerable investments.

Notwithstanding the above challenges, peer support networks keep popping up in many different contexts. From the perspective of full self-management, this is a positive trend, as peer support networks have a great potential to empower patients. They can improve knowledge, skills and attitudes of individual patients, but also contribute to mobilising and empowering patients as a group, for instance to demand better access and quality of treatment.

### Smart phone technology

The use of mobile phones is rapidly expanding, especially in the developing world [84,85]. The potential of the use of mobile phones and other information and communication technologies in health care is enormous [84]. Until now, the use of mobile phones in health care has been largely limited to improved communication between patients and health care providers. Mobile phones have been most commonly used in the care for chronic patients to support behaviour change, to transmit results and to send appointment reminders [86-88].

The use of mobile technology can be particularly beneficial for the management of chronic diseases, like diabetes, which are characterised by the need for behaviour change, long latency periods, interactions at different levels of the health care system and self-management [84].

Studies about mobile phone applications in health care in developing countries are increasingly published [84,89,90]. In Malawi, the provision of mobile phones to Community Health Workers (CHW) to use in their activities related to home-based care, tuberculosis, mother and child health and anti-retroviral treatment, for instance for reporting adherence, sending appointment reminders and asking questions to health professionals, turned out feasible, effective and cost-effective, especially in saving travel expenses [91]. Mobile phone intervention led to statistically significant improvements in glycaemic control and self-management in diabetes care [92].

Other applications of mobile phones, especially of smart phones, can drastically enlarge the possibilities of people with CLLC for full self-management. A smart phone is a mobile phone that has more advanced computing ability and communication options than a regular mobile phone [93]. It has, for instance, the possibility to store and read documents and to connect to the internet. The medical industry is increasingly developing diagnostic test devices that can be connected to smart phones, such as sphygmomanometers and glycometers, and software that interprets and records the results. Examples are smart phone programmes linked with glycometers that calculate the recommended dose of insulin based on the measured blood glucose [94]. Currently there still exist great inequities regarding the availability and affordability of this technology, but its development and accessibility will quickly expand in the coming years, especially as prices are expected to fall rapidly [95]. The combination of different technologies such as internet, software and diagnostic tools will further enlarge the possibilities.

The development and proliferation of smart phone technology drastically expand the possibilities for total self-management. The patients can perform diagnostic tests and receive the interpretation and subsequent treatment advice without any external help, and this anytime and anywhere. If necessary, they can easily share these results with others and ask for personal advice. These are very new developments and their use in health care has hardly been described so far. Yet, the example of the use of smart phones in a cardiac rehabilitation programme shows the feasibility and the potential of this technology, especially to create more flexibility for patients in coping [96].

### Ways forward

Our proposal for full self-management is still a working hypothesis, but in our view, it is one that definitely merits field testing. There should be space for creativity and flexibility, to explore the possibilities of different social media and communication tools, to develop information sites for low bandwidth, peer patient platforms at Facebook, text messages and twitter options for acute questions, etcetera. Existing internet-based self-management programmes could be an opportunity to start experimenting [97]. Various models should be explored, depending on context variables, such as the burden of disease, the availability of professional health staff, the capacity and quality of health services, the availability of tools and infrastructure, the spread of technologies, social transitions and other factors. They should be evaluated for their effect on empowerment and on health outcomes. The expertise and funding to develop and distribute these applications are likely to come not only from public sources, but also from private companies envisaging future markets. This might help to scale up interventions by stimulating supply and demand, but a strong regulatory framework is necessary to prevent interference of profit motives with public health objectives. The risk of increasing inequity by using technologies that are only accessible to the better off is real. Initiatives to bridge the digital divide, similar to the "one laptop per child" initiative, could contribute to addressing this inequity [98]. Experience shows that new media and smartphones can be easily mastered by people of all ages with very little education in deprived circumstances [99].

The governance challenge is to bring the different medical, social and technical developments together, and steer them towards delivery models for chronic care that are adjusted to the context. Well-functioning delivery models require that adequate medical care is given, that different actors in the delivery of care and support - peer networks, professional providers, lay workers - link up and work together and that technical applications such as mobile phone applications are accessible. The use of peer networks and smartphone technology can also benefit more professionalised models of care. There is not one uniform model or blueprint. However, in our view, the ultimate goal of adequate chronic care should be to empower people so that they become experts in managing their lives with their chronic condition, using all dimensions of support, networks and tools when necessary.

## List of abbreviations used

ART: Anti-Retroviral Treatment; CCM: Chronic Care Model; CLLC: Chronic Life-Long Conditions; LIC: Low Income Countries; NCD: Non-Communicable Diseases; US: United States.

## Competing interests

The authors declare that they have no competing interests.

## Authors' contributions

JVO, GMK, RB, GK, KH, WVD have all contributed to this paper. All authors have read and approved the final manuscript.
